# Urinary Metabolomic Profile of Neonates Born to Women with Gestational Diabetes Mellitus

**DOI:** 10.3390/metabo11110723

**Published:** 2021-10-22

**Authors:** Ana Sofía Herrera-Van Oostdam, Mariana Salgado-Bustamante, Victoria Lima-Rogel, Juan José Oropeza-Valdez, Jesús Adrián López, Iván Daniel Román Rodríguez, Juan Carlos Toro-Ortiz, David Alejandro Herrera-Van Oostdam, Yamilé López-Hernández, Joel Monárrez-Espino

**Affiliations:** 1 Centro de Investigación en Ciencias de la Salud y Biomedicina, Universidad Autónoma de San Luis Potosí, San Luis Potosí 78290, Mexico; sofiaherreravo@alumnos.uaslp.edu.mx; 2 Departamento de Bioquímica, Universidad Autónoma de San Luis Potosí, San Luis Potosí 78290, Mexico; mariana.salgado@uaslp.mx; 3 Hospital Central “Dr. Ignacio Morones Prieto”, San Luis Potosí 78290, Mexico; limamv@hotmail.com (V.L.-R.); jcarlostoro@yahoo.com.mx (J.C.T.-O.); davempkin@msn.com (D.A.H.-V.O.); 4 Instituto Mexicano del Seguro Social, Unidad de Investigación Biomédica de Zacatecas, Zacatecas 98000, Mexico; intrinsection@hotmail.com; 5 MicroRNAs and Cancer Laboratory, Unidad Académica de Ciencias Biológicas, Universidad Autónoma de Zacatecas, Zacatecas 98000, Mexico; jalopez@uaz.edu.mx; 6 Metabolomics and Proteomics Laboratory, Universidad Autónoma de Zacatecas, Zacatecas 98000, Mexico; ivandier03@gmail.com; 7 CONACyT, Metabolomics and Proteomics Laboratory, Autonomous University of Zacatecas, Zacatecas 98000, Mexico; 8 Department of Health Research, Christus Muguerza del Parque Hospital Chihuahua, University of Monterrey, San Pedro Garza García, Nuevo León 66238, Mexico

**Keywords:** newborns, metabolomics, gestational diabetes, pregnancy

## Abstract

Gestational diabetes mellitus (GDM) is one of the most frequent pregnancy complications with potential adverse outcomes for mothers and newborns. Its effects on the newborn appear during the neonatal period or early childhood. Therefore, an early diagnosis is crucial to prevent the development of chronic diseases later in adult life. In this study, the urinary metabolome of babies born to GDM mothers was characterized. In total, 144 neonatal and maternal (second and third trimesters of pregnancy) urinary samples were analyzed using targeted metabolomics, combining liquid chromatographic mass spectrometry (LC-MS/MS) and flow injection analysis mass spectrometry (FIA-MS/MS) techniques. We provide here the neonatal urinary concentration values of 101 metabolites for 26 newborns born to GDM mothers and 22 newborns born to healthy mothers. The univariate analysis of these metabolites revealed statistical differences in 11 metabolites. Multivariate analyses revealed a differential metabolic profile in newborns of GDM mothers characterized by dysregulation of acylcarnitines, amino acids, and polyamine metabolism. Levels of hexadecenoylcarnitine (C16:1) and spermine were also higher in newborns of GDM mothers. The maternal urinary metabolome revealed significant differences in butyric, isobutyric, and uric acid in the second and third trimesters of pregnancy. These metabolic alterations point to the impact of GDM in the neonatal period.

## 1. Introduction

Gestational diabetes mellitus (GDM), defined as hyperglycemia during gestation [1], is the most frequent medical complication in pregnancy, affecting 7–10% of all pregnancies worldwide [2]. GDM has been associated with various adverse outcomes both in mothers and newborns. Women with GDM are at higher risk of developing type 2 diabetes (T2D), mainly 3–6 years after delivery, and their offspring is also at higher risk of becoming overweight and obese [3]. The metabolic abnormalities associated with GDM include increased insulin resistance and β-cell defects, which can occur before conception, especially in populations with a high prevalence of obesity and T2D.

Pregnancy is a complex physiological condition associated with relevant metabolic changes resulting from increased energy and nutrition requirements needed for fetal development [4,5]. However, studies comparing the metabolic profiles of women with GDM and their offspring are still scarce.

There are only a few reports from newborn metabolome addressing the effect of maternal GDM. Most of these studies have been performed in serum from the umbilical cord [6,7,8]. The first study on this topic reported that the metabolomic profile observed in serum from the umbilical cord of neonates born to GDM women differed between those with and without pregnancy complications, suggesting that a prolonged fetal exposure to hyperglycemia could impact the newborn’s metabolome [4]. In cord blood, Lu et al. found that fetal phosphatidylcholine acyl-alkyl C 32:1 and proline showed an independent association with GDM [8]. In a recent work, associations between GDM and higher offspring ketone levels in cord serum at birth were found consistent with maternal ketosis in diabetic pregnancies [9]. Only a study using untargeted metabolomics compared urine and meconium between neonates born to GDM women and healthy controls, finding metabolic differences. However, in this study, no maternal urine or serum samples were available for analyses [5].

In the present work, urine samples were collected from 48 full-term healthy neonates born to GDM mothers and controls. Urine samples were also collected from mothers in the second and third trimesters of pregnancy to investigate whether metabolic alterations found in babies could relate to maternal metabolic alterations. All the samples were analyzed using targeted MS/MS-based metabolomic assays to assess the potential influence of GDM in the early urinary metabolome of newborns. Multivariate analyses revealed a differential metabolic profile in newborns of GDM mothers characterized by the dysregulation of long-chain acylcarnitines, amino acids, short-chain fatty acids, and polyamine metabolism. A dysregulation in the arginine/nitric oxide (Arg/NO) signaling pathway, intestinal dysbiosis, or diet/treatment differences could explain these metabolic alterations. In addition, quantitative values of 101 metabolites measured in newborn urine samples collected within 24 h of birth are presented. Knowing these concentration values is of pivotal importance for clinicians and neonatologists to monitor the impact of GDM in the early stages of life.

## 2. Results

### 2.1. Clinical Characteristics of Newborns and Mothers

Comparisons of selected characteristics between mothers and newborns by diabetic status of the mother are shown in Table 1. Newborn gestational age, Apgar scores, and birth weight were similar between those born to GDM and healthy women. Women with GDM were 2.8 years older (*p* = 0.05). The prevalence of overweight was high in both groups (GDM 38.4%, healthy 40.9%; *p* = 0.32). Mean glucose measured at the first prenatal visit was also 7.1 mg/dL higher in women with GDM (*p* = 0.03). From all GDM women, 15 received metformin and one received combined treatment for diabetes control. All women with GDM were diagnosed between weeks 24 and 28 of gestation.

### 2.2. Urinary Concentration Values of 101 Metabolites of Newborns to Diabetic and Healthy Mothers

From the 136 metabolites analyzed, 101 were absolutely quantified (35 metabolites belonging to glycerophospholipid and sphingomyelin classes were excluded from the statistical analysis as they fell below the limit of detection by the method employed). Table 2 shows the concentration values of the statistically significant metabolites quantified, both for babies born to GDM and healthy mothers. Supplementary Table S1 shows the concentration levels for the rest of measured metabolites. Mann–Whitney U tests revealed that 11 metabolites were statistically different between the study groups (trans-hydroxyproline, glutamic acid, DOPA, spermine, lactic acid, butyric acid, isobutyric acid, C5:1DC, C5DC, C10:2, and C16:1).

### 2.3. Multivariate Analysis

**Figure 1:**
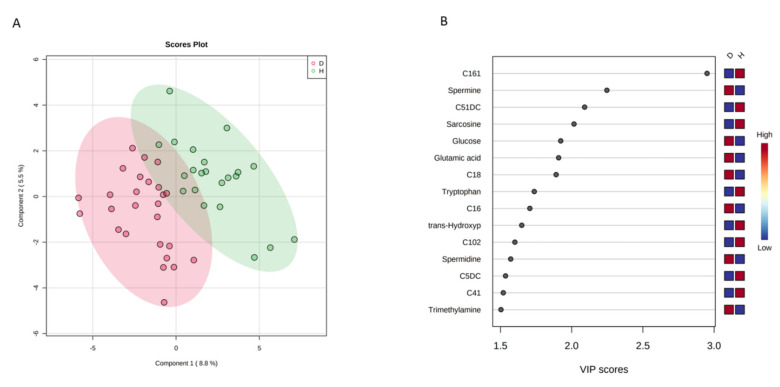
Multivariate analysis showing the comparison between urine metabolite data acquired for newborns of GDM (D) and healthy mothers (H). (**A**) Partial least squares discriminant analysis (2-D PLS-DA) score plots; (**B**) Variable importance in projection plot. The most discriminating metabolites are shown in descending order of their coefficient scores.

Figure 1 shows the results from multivariate analyses performed to compare the newborns of GDM mothers with those from healthy mothers. Supervised PLS-DA (Figure 1A) showed a partial separation with low risk of overfitting (*p* = 0.008 for 2000 permutation test). Figure 1B shows the variable importance in projection for 15 metabolites with a VIP >1.5. C16:1 and spermine were the most discriminant metabolites and remained significant also after FDR adjustment (q < 0.05).

When these metabolites were modeled, adjusting by mother’s age in years and pre-pregnancy BMI in kg/m^2^, only spermine remained significant (*p* < 0.05). Such model had a non-significant Hosmer–Lemeshow Chi^2^ *p*-value of 0.30, indicating a good fit, and a Nagelkerke R^2^ of 0.62, indicating that the model explained 62% of the variability of the outcome (i.e., diabetes status of the mother). 

**Figure 2:**
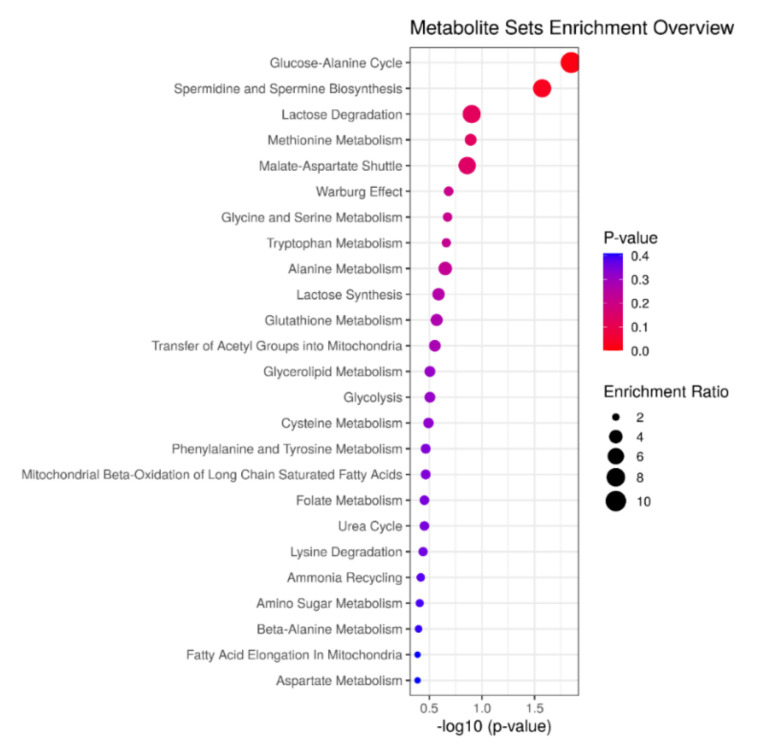
Pathway analysis of the deregulated metabolites in newborns to GDM mothers. The node size is proportional to the enrichment ratio.

The pathway-enrichment analysis revealed that discriminant metabolites were mainly involved in amino acid, carbohydrate, spermine, and spermidine biosynthesis and lipid metabolism-related pathways, as shown in Figure 2.

### 2.4. Analysis of the Maternal Urinary Metabolome during the Second and Third Trimesters of Pregnancy

To assess the possible relation between the metabolic alterations found in the newborns and those of their mothers, maternal urine samples collected during the second half of the pregnancy were analyzed.

**Figure 3:**
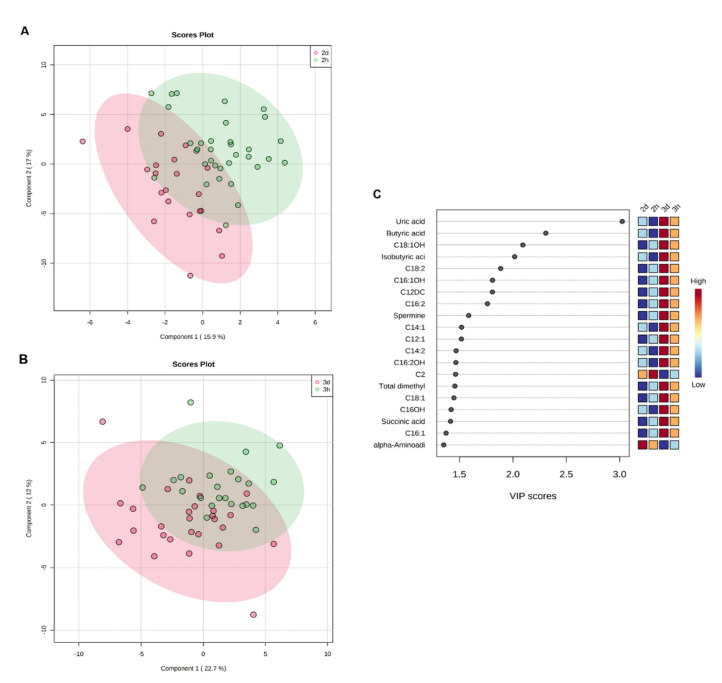
Multivariate analysis showing the comparison between urinary metabolite data acquired for GDM and healthy mothers in the second and third trimesters, respectively. (**A**) Partial least squares discriminant analysis score plots (2-D PLS-DA) of healthy pregnant women with GDM during the second trimester of gestation. (**B**) 2-D PLS-DA of healthy pregnant women with GDM during the third trimester of gestation. (**C**) Importance of the variable in the projection graph. The most discriminating metabolites are shown in descending order of their coefficient scores. Second trimester of gestation with GDM (2 d); third trimester of gestation with GDM (3 d); healthy second trimester of pregnancy (2 h); healthy third trimester of pregnancy (3 h).

Multivariate analyses did not show a clear separation between healthy and GDM mothers in the second (Figure 3A) or third (Figure 3B) trimesters. VIP analysis (Figure 3C) shows the top 20 metabolites differentially expressed between healthy and GDM mothers in their second and third trimesters. Most of the metabolites altered in GDM newborns were also increased in GDM mothers in the third trimester. However, only isobutyric, butyric, and uric acid were significantly dysregulated between GDM and healthy mothers, and between trimesters of gestation (q < 0.05).

## 3. Discussion

This study aimed at analyzing the urinary metabolome of babies born to GDM and healthy mothers. To our knowledge, this is the first work reporting quantitative concentration values for 101 metabolites measured in urine within the first 24 h of life of neonates born to GDM mothers. Since there are no previous studies presenting quantitative analyses performed on the urine of newborns of GDM mothers, comparison with these findings was not possible. Despite most urinary metabolite concentrations of all newborns included in the present work being within the normal range, as documented earlier [10], our results showed a differential profile in the urinary levels of some amino acids, polyamines, and carnitines among newborns of GDM mothers.

While we found 11 differential metabolites in the neonatal urine, after the Benjamini Hochberg (BH) procedure, only two metabolites remained as significant. Spermine was increased in the urine of babies born to GDM mothers. The higher levels of spermine in GDM babies could be a result of a dysregulation in the arginine/nitric oxide (Arg/NO) signaling pathway. GDM has been associated with endothelial dysfunction due to a dysregulated endothelial Arg/NO signaling pathway [11]. The activity of this signaling pathway is modulated by D-glucose, adenosine, insulin, and ATP, among other molecules in T2D, GDM, and other diseases coursing with vascular and/or endothelial compromise [12]. An increase in the level of serum arginine in the umbilical artery of women with GDM has been reported, suggesting that GDM upregulates the Arg/NO pathway. This could, in turn, explain the high levels of spermine seen in this and other studies, since the arginine pathway is involved in the polyamine synthesis [11]. Previously, it was found that increased adiposity in children was associated with an increase in the three circulating polyamine levels, including spermine [13]. The authors showed that the spermine level was related to markers of the NO pathway, oxidative stress, inflammation, and leptin [13]. Polyamine metabolism has been involved in adipogenesis, suggesting that increased polyamine levels may be implicated in adipose tissue expandability during obesity. A close relationship has also been proposed between polyamine levels and the gut microbiota composition. In fact, the gut microbiota area considered as mainly responsible for polyamine levels in the lower part of the intestine from where they are transferred into the bloodstream via the colonic mucosa [14]. Polyamines can also be acquired by breast milk during lactation, although Atiya Ali et al. found that the spermine levels did not differ between breast milk from obese mothers and mothers with normal body weight [15]. The results presented here suggest that spermine could be monitored during the neonatal period and childhood due to its proven relation with metabolic disorders such as obesity.

A significant decrease of urinary C16:1 in babies born to GDM mothers was also observed. In GDM pregnancies, data on carnitines are scarce, but increased carnitine levels in GDM mothers and their offspring have been reported without clear explanation [16]. Shokry et al. found that reduced levels of C16:0 in GDM was associated with diminished placental transport of NEFAs and carnitines along with incomplete or reduced FAO, which was reported in insulin resistance and T2D [7]. Several studies have reported an increase in the concentration of long-chain carnitines (C12–C16) in plasma and urine samples of T2D patients, suggesting an incomplete oxidation of long-chain fatty acids, altered activity of the tricarboxylic acid cycle, and an increased flow of fatty acids to the mitochondria, which are molecular mechanisms that contribute to the pathogenesis of insulin resistance [17,18,19]. Some authors have proposed a similar mechanism for GDM. Lin et al. evaluated the association of plasma acylcarnitine profiles and GDM throughout gestation, reporting that elevated levels of C4, C8:1, and C16:1-OH were associated with an increased risk of GDM [20]. Batchuluun et al. found a specific elevation in the serum of hexanoylcarnitine and octanoylcarnitine among women with GDM and individuals with T2D without alteration in long-chain acylcarnitines [21]. In contrast, Pappa et al. reported for the first time that GDM does not further affect the efficiency of the carnitine system. The authors proposed that the mild effect of GDM on carnitine status could be explained by the concurrent increased gluconeogenesis, a process that does not directly affect carnitine metabolism [16]. The decrease of only one long acylcarnitine in our study is insufficient to suggest one possible molecular mechanism. Additional studies would be needed to propose a clear explanation for this C16:1 decrease. Although the trend for urinary concentrations of long acylcarnitines was in general to decrease (Supplementary Table S1), no significant differences were found except for C16:1. In line with our results, Sánchez-Pinto et al. compared GDM and no GDM Large for Gestational Age (LGA) newborns, finding that a history of GDM was associated with lower levels of medium- and long-chain acylcarnitines, although the differences were not significant. From the analysis of the individual acylcarnitines, no remarkable differences were revealed [22].

The urinary metabolome in the second and third trimesters of pregnancy was studied to explore the potential impact of maternal alterations on the newborn urinary metabolome. The study presented here compared urinary metabolites between healthy and GDM women in the second and third trimesters. A distinctive pattern between GDM and healthy pregnant women was not observed. This is in general agreement with what other researchers have reported. For instance, an earlier longitudinal study using a non-targeted approach found that urine metabolites associated with GDM could be detected in the first trimester, but not in the third trimester [23]. In this study, nearly all women diagnosed with GDM were treated with insulin or metformin and dietary/lifestyle interventions to bring their condition under control. This suggests that the urinary metabolic profile of GDM patients could be substantially affected by medical and/or dietary interventions. Similar to that observed here, a large multiethnic study showed that while changes in the maternal urinary profile could be seen during and after pregnancy, no identifiable and reliable biomarkers of GDM were seen [24]. Another study did find alterations in the urinary excretion of some amino acids, but failed to correlate well with the glycemic control of GDM women [25]. Finally, a non-targeted UPLC-MS characterization of second trimester maternal urine and amniotic fluid was unable to find associated changes in the pre-diagnostic GDM group [26]. Overall, the effect of pharmacological treatment (metformin, insulin, or combination of both), diet, and exercise, appears to normalize the metabolic profile of GDM patients to match (or nearly match) that of healthy controls in the second and third trimesters of pregnancy.

Interestingly, levels of butyric and isobutyric acids (SCFAs and BSCFAs, respectively) increased significantly between the second and third trimesters in both healthy and GDM women in this study. This replicates a finding that reported an increase in butanoate metabolism throughout pregnancy in urine samples of GDM women [27] and serum [6].

SCFAs are produced through colonic fermentation of dietary fibers, while BSCFAs are generated from undigested protein reaching the colon and are associated with the fermentation of branched amino acids [28]. In this study, a large proportion (38.5%) of GDM women were overweight (BMI > 25 kg/m^2^). In overweight women with GDM, a shift in the microbiota composition to higher α-diversity has been observed, along with numerous associations between metabolic and inflammatory patterns and specific bacterial abundance [29]. For instance, in a study with obese Mexican women, with and without metabolic syndrome, firmicutes bacteria were the most abundant gut bacterial phylum [30]. While bacterial presence was not determined in this population, excessive production of SCFA (butyrate) and BSCFA (isobutyrate) was found among GDM women in the second and third trimesters. It has been suggested that there is an obesity-associated gut microbiota that harvests more energy from soluble dietary fiber through fermentation and produces more short-chain fatty acids than lean individuals, influencing host energy metabolism [30]. Butyric and isobutyric acid were also found to be dysregulated in the GDM newborn urinary metabolome, which constitutes a possible connection between maternal and newborn metabolomes influenced by GDM. In addition, intestinal dysbiosis (indirectly reflected by altered levels of butyric and isobutyric acid) can also affect the pool of polyamine metabolism including spermine levels [31].

This study has a number of strengths and weaknesses that need to be highlighted. The application of quantitative, targeted metabolomics to study urinary metabolites of babies born to GDM mothers makes it valuable for clinicians and neonatologists, helping in the screening of the early newborn metabolism. On the other hand, the main limitations relate to the relatively small sample size and the uncontrolled follow-up of dietary regimen in GDM mothers, which may also modulate the maternal metabolome. This led to relatively large standard errors, imprecise estimates, and to potential selection bias. In spite of the fact that urine is a biofluid with a high content of metabolites and its collection is non-invasive, a number of relevant metabolites for the GDM physiopathology such as lipids had to be excluded because they were below the detection limit for the method employed. Future research is needed to confirm the findings reported here and to address these study limitations.

## 4. Materials and Methods

### 4.1. Study Design and Research Ethics Approval

**Figure 4:**
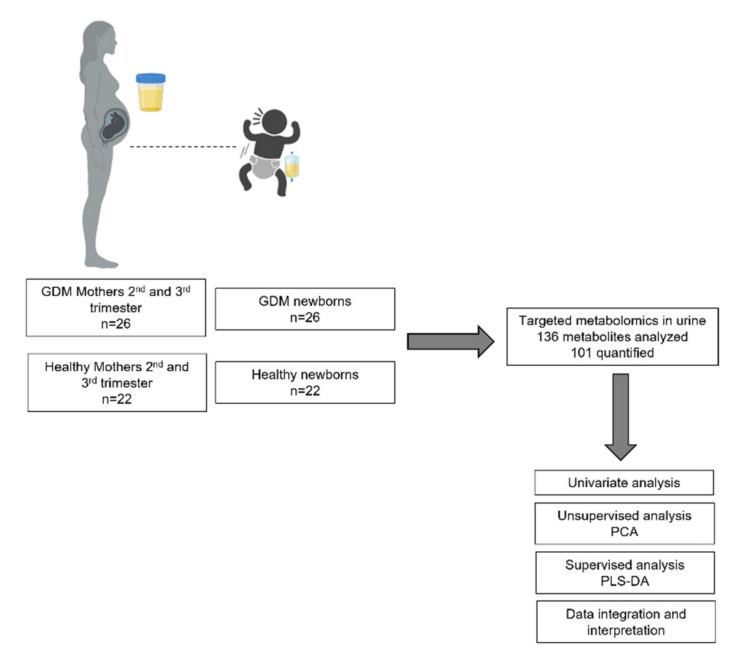
Study design: Longitudinal study of 48 mother–newborn pairs.

The study was carried out between May 2018 and April 2020 at the Hospital Central “Dr. Ignacio Morones Prieto” from San Luis Potosi, in Mexico. The study design is summarized in Figure 4. The research proposal was revised and approved by the Hospital’s Research and Ethics Committee (Registration No. 84-17; CONBIOETICA-24-CEI-001-201604279).

All relevant ethical aspects were carefully considered, and the Helsinki Declaration was followed. Written informed consent was signed by all participant women prior to the interview and to urine sample collection including that of their babies.

### 4.2. Study Population

*Newborns:* Forty-eight newborns were examined by a neonatologist (26 newborns of mothers with GDM and 22 of healthy mothers). Collected data included sex, weight, type of delivery, gestational age, APGAR score (i.e., appearance, pulse, grimace, activity, and respiration at minutes 1 and 5), Capurro test, and Silverman–Anderson score (Table 1). The first urine collection from babies born to GDM and healthy women was carried out within the 24 h after birth once the babies’ genitals were thoroughly cleaned, placing the samples into sterile bags until micturition. Samples contaminated with meconium were discarded.

*Mothers:* Forty-eight pregnant women were recruited. The GDM group was composed of 26 patients who were diagnosed with GDM during the second trimester. The control group included 22 euglycemic women. GDM diagnosis was based on a positive oral glucose tolerance test undertaken between weeks 24 and 28 using the diagnostic criteria established by the WHO and the American College of Obstetricians and Gynecologists. Pregnant women with hypertension or preeclampsia, T2D, urinary infections, chronic renal disease, cancer, or polycystic ovary syndrome were excluded. All GDM patients were given treatment after diagnosis until delivery. In addition to diet and moderate exercise counseling, patients were prescribed metformin, insulin, or a combination of both. Clinical and demographic data were collected from medical records for each participant at the first prenatal visit. A total of 96 urine samples (first-morning urine) from the 48 pregnant women was collected between weeks 24 and 28 (first sample, second trimester), and weeks 30 and 34 (second sample, third trimester) of gestation.

### 4.3. Metabolite Measurements

A targeted quantitative metabolomics assay called The Metabolomics Innovation Center (TMIC) Prime (TMIC PRIME^®^) Assay was employed using a combination of direct injection (FIA) MS and reverse-phase high-performance liquid chromatography tandem mass spectrometry (LC-MS/MS). The method was previously described by Zheng et al. [32]. Samples were derivatized prior to MS analysis. Amino acids, biogenic amines and derivatives, acylcarnitines, lipids, and glucose were derivatized with phenylisothiocyanate (PITC), while for organic acids, 3-nitrophenylhydrazine, 1-ethyl-3-(3-dimethylaminopropyl) carbodiimide, and pyridine were added to achieve derivatization.

LC-MS/MS was used for the analysis of amino acids, biogenic amines and derivatives, and organic acids. An Agilent reversed-phase Zorbax Eclipse XDB C18 column (3.0 mm × 100 mm, 3.5 μm particle size, 80 Å pore size) with a Phenomenex (Torrance, CA, USA) SecurityGuard C18 pre-column (4.0 mm × 3.0 mm) was used. The LC and MS parameters are described elsewhere [10]. Seven-point calibration curve was generated for each analyte.

The FIA-MS/MS method was employed for the analysis of lipids, acylcarnitines, and glucose; the LC autosampler was connected directly to the MS ion source by red PEEK tubing. Lipids, acylcarnitines, and glucose were analyzed semi-quantitatively.

### 4.4. Statistical Analysis

Means and standard deviations (s.d.) or medians with interquartile range (IQR) and percentiles were calculated for continuous data with normal and non-normally distributed data, respectively. Normality of the distributions was assessed by the Kolmogorov–Smirnov test. GraphPad Prism 7 software (GraphPad Software, Inc., La Jolla, CA, USA) was used.

Statistical procedures were conducted as described elsewhere for quantitative metabolomics [33]. Univariate analyses were performed using Mann–Whitney rank sum tests and Fisher’s exact tests, while principal component analysis (PCA) and partial least squares discriminant analysis (PLS-DA) were conducted using MetaboAnalyst [34]. A 2000-fold permutation test was performed to assess the significance. The differential variables were selected according to three conditions: (1) adjusted *p* < 0.05; (2) fold change between two groups >1.5; and (3) variable importance for the projection (VIP) value obtained from PLS-DA >1.5. Metabolic pathway analysis was carried out using the pathway analysis module of MetaboAnalyst 4.0. The Benjamini and Hochberg method was used to adjust the *p* values. This method, rather than controlling the false positive rate, controls the false discovery rate (FDR). In the FDR method, *p* values are ranked in an ascending array and multiplied by m/k where k is the position of a *p* value in the sorted vector and m is the number of independent tests [35].

## 5. Conclusions

In this study, an extensive quantitative characterization of the urinary metabolome of newborns to GDM and healthy mothers is presented. Concentration values of 101 metabolites detected in the urine of newborns collected within the first 24 h of life were available for analyses. Spermine and hexadecenoylcarnitine were dysregulated in newborns of GDM mothers. Additionally, urinary maternal samples from the second half of pregnancy were used to assess the influence of GDM in the urinary metabolome of newborns. GDM mothers in the second and third trimester of gestation had increased urinary levels of isobutyric, butyric acid, and uric acid. Apparently, GDM slightly modulates the maternal metabolome, with altered levels of butyric and isobutyric acid, which are also detected in the urinary metabolome of newborns. This information is of clinical relevance for neonatologists and pediatricians to monitor the impact of GDM in the early stages of life.

## Figures and Tables

**Table 1:** Comparison of selected characteristics of the pregnant women and their newborns by diabetes status of the mother.

Characteristics	GDM	Healthy	*p*-Value *
**Newborns, n (%)**	26 (54.1)	22 (45.8)	
Sex, n (%) ^b^			
Female	16 (61.5)	4 (18.1)	0.003 **
Male	10 (38.4)	18 (81.8)	
Gestational age (weeks) ^a^	38.5 ± 1.3	38.3 ± 1.2	0.5
APGAR score, min 1 ^b^	8 (100.0)	8 (100.0)	1.00
APGAR score, min 5 ^b^	9 (100.0)	9 (100.0)	1.00
Silverman-Anderson score ^b^	0 (100.0)	0 (100.0)	1.00
Weight (g) ^a^	3026 ± 399	2943 ± 477	0.4
Delivery, n (%) ^b^			
Vaginal	9 (34.6)	13 (59.1)	0.1
C-section	17 (65.4)	9 (40.9)	
**Mothers, n (%)**	26 (54.1)	22 (45.8)	
Age (years) ^a^	28.4 ± 4.7	25.6 ± 2.2	0.05
Pre-BMI (Kg/m^2^) ^a^	27.87 ± 4.12	25.58 ± 4.22	0.06
Normal weight, n (%) ^b^	8 (30.8)	10 (45.5)	0.3
Overweight, n (%) ^b^	10 (38.5)	9 (40.9)	0.3
Obese, n (%) ^b^	8 (30.8)	3 (13.6)	0.3
Glucose (mg/dL) ^a^	86.94 ± 13.3	79.81 ± 8.7	0.03 *
Creatinine (mg/dL) ^a^	0.56 ± 0.08	0.57 ± 0.09	0.6
Urea (mg/dL) ^a^	14.26 ± 4.0	14.19 ± 3.91	1.0
Hemoglobin (g/dL) ^a^	13.02 ± 1.0	12.81 ± 0.75	0.4
Leucocytes (×10^3^) ^a^	9.01 ± 2.65	8.34 ± 1.70	0.3
SBP (mm Hg) ^a^	113.1 ± 8.7	108.2 ± 9.6	0.07
DBP (mm Hg) ^a^	74.23 ± 7.02	72.73 ± 7.67	0.5
Treatment, n (%)			
Metformin	15 (57.7)		
Diet and exercise	10 (38.5)		
Insulin + Metformin	1 (3.8)		

Pre-BMI: prenatal body mass index; SBP: systolic blood pressure; DBP: diastolic blood pressure. * *p* value from Student *t*-tests were used for normally distributed and Mann–Whitney U tests for non-normally distributed continuous variables, * *p* ≤ 0.05, ** *p* ≤ 0.01; Chi^2^ tests and Fisher’s exact tests were used for nominal data. ^a^ Student’s *t* test (mean ± S.D), ^b^ Chi square or Fisher’s exact tests.

**Table 2:** Concentration values of statistically significant metabolites measured for babies born to mothers with GDM and healthy controls.

Metabolite	Healthy Newborns	GDM Newborns	*p* Value
	Median (2.5–97.5 IQR) (μM/mM Creatinine)	Median (2.5–97.5 IQR) (μM/mM Creatinine)	
trans-Hydroxyproline	36.0 (8.1–96.65)	26.4 (11.31–60.82)	0.01
Glutamic acid	7.4 (1.88–25.28)	12.4 (3.72–36.52)	0.01
DOPA	0.06 (0.02–0.17)	0.04 (0.01–0.08)	0.04
Spermine	0.03 (0.007–0.09)	0.04 (0.006–0.72)	0.003 *
Lactic acid	85.6 (46.95–797.5)	112.0 (47.14–359.8)	0.04
Butyric acid	0.33 (0.12–0.9)	0.22 (0.06–0.7)	0.02
Isobutyric acid	0.08 (0.03–1.0)	0.05 (0.02–1.0)	0.03
Glutaconylcarnitine (C5:1DC)	0.02 (0.008–0.03)	0.01 (0.005–0.03)	0.009 *
Glutarylcarnitine (C5DC)	0.05 (0.03–0.1)	0.04 (0.02–0.06)	0.006 *
C10:2	0.03 (0.02–0.07)	0.03 (0.014–0.05)	0.01
Hexadecenoylcarnitine (C16:1)	0.012 (0.007–0.03)	0.009 (0.004–0.027)	0.01

*p* < 0.05 was considered statistically significant; * *p* < 0.01.

## Data Availability

The data presented in this study are available in Table S1: Concentration values of the metabolites measured for babies born to mothers with GDM and healthy controls.

## Supplementary Materials

The following are available online at https://www.mdpi.com/2218-1989/11/11/723/s1, Table S1: Concentration values of the metabolites measured for babies born to mothers with GDM and healthy controls.
