# Supplementary material for: Urinary Metabolomic Profile of Neonates Born to Women with Gestational Diabetes Mellitus
Source: Metabolites. 2021 Oct 22;11(11):723. doi: 10.3390/metabo11110723 (PMC8621167; doi:10.3390/metabo11110723)
Supplement: Supplementary file 1 [file metabolites-11-00723-s001.zip › metabolites-1422648-supplementary.pdf]

Table S1: Concentration values of the metabolites measured for babies born to mothers with GDM and healthy controls.

Metabolite	Healthy newborns median (2.5 -97.5 IQR) (μM/mM creatinine)	GDM newborns median (2.5 -97.5 IQR) (μM/mM creatinine)	P value
Glycine	356.9 (101.8-879.5)	257.0 (105.5-747.6)	0.22
Alanine	83.8 (31.78-153.4)	66.7 (37.08-125.2)	0.34
Serine	78.4 (31.4-126.0)	68.0 (47.51-149.4)	0.51
Histamine	0.02 (0.0073-0.041)	0.02 (0.008-0.041)	0.08
Proline	22.4 (9.511-63.78)	24.4 (9.49-82.39)	0.68
Valine	5.7 (0.74-16.98)	7.032 (1.87-18.86)	0.08
Threonine	26.0 (9.1-99.45)	30.0 (15.16-84.91)	0.39
Taurine	924.6 (69.86-2 851)	1118 (91.11-2 809)	0.38
Putrescine	0.14 (0.03-3.68)	0.11 (0.05-1.14)	0.36
Leucine	4.4 (0.55-13.53)	6.3 (0.26-12.58)	0.14
Isoleucine	2.3 (0.23-6.1)	2.8 (0.5-7.7)	0.04*
Asparagine	3.3 (0.36-16.9)	2.8 (1.06-15.24)	0.78
Aspartic acid	17.0 (6.26-33.0)	17.2 (5.37-34.65)	0.95
Glutamine	50.2 (15.47-129.3)	50.8 (24.58-135.8)	0.73
Methionine	1.9 (0.65-4.96)	2.4 (0.51-4.3)	0.20
Dopamine	0.73 (0.13-2.14)	1.33 (0.18-2.63)	0.06
Histidine	61.8 (18.7-193.6)	48.4 (29.2-169.2)	0.51
alpha-Aminoadipic acid	6.6 (1.3-21.3)	6.7 (1.2-691.2)	0.71
Phenylalanine	6.7 (4.1-14.7)	7.3 (4.4-12.4)	0.32
Methionine-sulfoxide	1.6 (0.6-2.9)	1.4 (0.6-3.6)	0.84
Arginine	14.6 (7.2-30.9)	13.3 (6.7-29.2)	0.79
Acetyl-ornithine	0.42 (0.12-3.3)	0.42 (0.14-3.5)	0.51
Serotonin	0.17 (0.12-0.3)	0.2 (0.1-0.36)	0.23
Tyrosine	6.2 (2.08-15.11)	5.0 (2.15-10.25)	0.34
Asymmetric dimethylarginine	2.2 (0.972-5.03)	2.3(1.4-4.327)	0.65
Total dimethylarginine	11.0 (6.3-17.82)	11.5 (6.84-21.3)	0.49
Tryptophan	3.32 (1.88-6.17)	3.24 (1.85-5.54)	0.57
Kynurenine	0.1 (0.03-0.72)	0.1 (0.02-0.68)	0.26
Carnosine	3.05 (0.45-36.23)	2.45 (0.56-21.87)	0.76
Ornithine	5.7 (1.28-25.5)	4.7 (2.03-20.5)	0.53

Lysine	52.3 (14.91-410.0)	43.9 (10.13-306.7)	0.76
Spermidine	0.04 (0.02-0.19)	0.06 (0.01-0.32)	0.06
Sarcosine	2.5 (0.67-11.36)	1.8 (0.51-5.194)	0.07
Diacetylspermine	0.9 (0.37-3.37)	0.9 (0.48-2.1)	0.97
Tyramine	0.9 (0.35-2.7)	0.9 (0.28-1.85)	0.57
Creatine	15.9 (4.63-455.8)	13.2 (5.9-241.5)	0.46
Betaine	221.3 (99.9-440.5)	168.8 (72.5-265.7)	0.05
Choline	16.4 (4.0-188.1)	16.4 (4.5-305.4)	0.36
Trimethylamine N-oxide	8.12 (0.08-45.23)	11.76 (1.32-33.0)	0.09
Methylhistidine	50.4 (17.31-146.8)	48.5 (22.81-139.9)	0.68
Beta-Hydroxybutyric acid	4.33 (0.71-45.0)	4.02 (0.78-38.0)	0.97
alpha-Ketoglutaric acid	123.4 (32.80-413.8)	81.0 (30.21-331.1)	0.60
Citric acid	395.0 (41.56-909.1)	391.0 (39.04-796.8)	0.34
p-Hydroxyhippuric acid	7.4 (4.2-19.96)	7.5 (4.7-18.33)	0.87
Succinic acid	17.8 (6.7-85.2)	16.9 (4.0-43.14)	0.94
Fumaric acid	13.55 (2.05-34.0)	11.13 (2.25-37.7)	0.55
Pyruvic acid	36.0 (19.3-93.0)	33.3 (22.72-63.5)	0.25
Hippuric acid	22.0 (1.3-156.6)	60.0 (4.8-213.6)	0.13
Methylmalonic acid	1.9 (0.42-22.51)	1.3 (0.42-6.64)	0.30
Homovanillic acid	0.7 (0.04-27.4)	0.5 (0.06-4.04)	0.58
Indole acetic acid	0.3 (0.018-1.98)	0.3 (0.08-1.182)	0.81
Uric acid	1622 (534.8-3 698)	1360 (174.6-4 333)	0.31
Glucose	385.5 (94.56-1111)	395.9 (248.3-2159)	0.25
Carnitine (C0)	1.7 (0.8-5.9)	1.8 (0.8-4.4)	0.25
L-Acetylcarnitine (C2)	0.8 (0.4-1.2)	0.9 (0.5-1.87)	0.09
Propenoylcarnitine (C3:1)	0.02 (0.004-0.06)	0.01 (0.01-0.05)	0.22
Propionylcarnitine (C3)	0.03 (0.013-0.07)	0.03 (0.02-0.07)	0.66
Butenylcarnitine (C4:1)	0.03 (0.01-0.04)	0.019 (0.008-0.04)	0.12
Butyrylcarnitine (C4)	0.1 (0.05-0.4)	0.1 (0.05-0.2)	0.49
Hydroxypropionyl carnitine (C3OH)	0.03 (0.009-0.07)	0.02 (0.007-0.05)	0.14
Tiglylcarnitine (C5:1)	0.06 (0.042-0.17)	0.07 (0.028-0.122)	0.84
Valerylcarnitine (C5)	0.06 (0.02-0.3)	0.05 (0.020-0.253)	0.83
Hydroxybutyryl carnitine (C4OH)	0.05 (0.013-0.08)	0.04 (0.018-0.077)	0.328
Hexenoylcarnitine (C6:1)	0.01 (0.007-0.02)	0.01 (0.007-0.022)	0.42

Hexanoylcarnitine (C6)	0.03 (0.017-0.04)	0.03 (0.015-0.05)	0.78
Hydroxyvalerylcarnitine (C5OH)	0.08 (0.04-0.33)	0.06 (0.05-0.154)	0.12
Octanoylcarnitine (C8)	0.04 (0.02-0.1)	0.04 (0.02-0.4)	0.49
Methylglutarylcarnitine (C5MDC)	0.06 (0.03-0.13)	0.05 (0.03-0.11)	0.45
Nonaylcarnitine (C9)	0.09 (0.05-0.19)	0.09 (0.03-0.15)	0.35
Pimelylcarnitine (C7DC)	0.05 (0.02-0.09)	0.042 (0.02-0.09)	0.30
Decenoylcarnitine (C10:1)	0.08 (0.05-0.2)	0.07 (0.033-0.19)	0.12
Decanoylcarnitine (C10)	0.07 (0.04-0.08)	0.06 (0.04-0.14)	0.30
Dodecenoylcarnitine (C12:1)	0.06 (0.03-0.14)	0.06 (0.02-0.12)	0.73
Dodecanoylcarnitine (C12)	0.07 (0.05-0.14)	0.07 (0.03-0.28)	0.76
Tetradecadienyl carnitine (C14:2)	0.01 (0.005-0.02)	0.01 (0.006-0.02)	0.92
Tetradecenoylcarnitine (C14:1)	0.009 (0.004-0.1)	0.01 (0.005-0.05)	0.49
Tetradecanoylcarnitine (C14)	0.019 (0.01-0.09)	0.019 (0.01-0.07)	0.64
C12DC	0.04 (0.015-0.33)	0.04 (0.02-0.15)	0.75
Hydroxytetradecadienylcarnitine (C14:2OH)	0.007 (0.004-0.012)	0.006 (0.003-0.015)	0.41
Hydroxytetradecenoyl carnitine (C14:1OH)	0.007 (0.004-0.01)	0.008 (0.004-0.015)	0.43
Hexadecadienyl carnitine (C16:2)	0.004 (0.003-0.011)	0.004 (0.002-0.007)	0.13
Hexadecanoylcarnitine (C16)	0.01 (0.006-0.02)	0.011 (0.006-0.071)	0.27
Hydroxyhexadecadienylcarnitine (C16:2OH)	0.004 (0.002-0.007)	0.003 (0.002-0.007)	0.19
Hydroxyhexadecenoyl carnitine (C16:1OH)	0.008 (0.005-0.015)	0.008 (0.004-0.03)	0.45
C16OH	0.008 (0.005-0.012)	0.007 (0.002-0.017)	0.60
Octadecadienylcarnitine (C18:2)	0.003 (0.001-0.007)	0.0020 (0.001-0.006)	0.18
Octadecenoylcarnitine (C18:1)	0.005 (0.003-0.01)	0.0042 (0.002-0.03)	0.30
Octadecanoylcarnitine (C18)	0.005 (0.002-0.01)	0.007 (0.002-0.08)	0.14
C18:1OH	0.005 (0.003-0.008)	0.004 (0.002-0.011)	0.17
